# Emasculation for Criminal Assaults and for Incest

**Published:** 1907-03

**Authors:** 


### EMASCULATION FOR CRIMINAL ASSAULTS AND FOR INCEST.

A bill is before the Texas Legislature providing for the castration of the rapist, for assault to rape, and for incest. It was introduced in the House by Mr. Walter of Gonzales, reported favorably, and it will in all probability pass the lower House, at least. The constitutional prohibition against "cruel and unusual punishment" can be obviated, it is thought, by showing that castration is to be inflicted (in addition to the penitentiary sentence), not as a punishment, but as a sanitary or hygienic measure to prevent a repetition of the offense, in the interest of public morals and race integrity, and the propagation of a race of sexual perverts—for the propensity on the part of a negro adult to rape a small white child is a perversion—and doubtless could be transmitted.

The bill provides that the court upon conviction, shall direct the sheriff of the county where the offense is committed, to cause the convict to be castrated by two surgeons of high character and standing, before taking him to the penitentiary. The moral effect on the community will be salutary, and no doubt will act as a powerful restraint on the evil-inclined, for a negro values thôse possessions more than life, and experience in Texas has shown that hanging and even burning by a mob does not restrain him. It will be a big step in the advance of civilization if we can get such a law in every State.

